# The overexpression of GDNF in nucleus accumbens suppresses alcohol-seeking behavior in group-housed C57Bl/6J female mice

**DOI:** 10.1186/s12929-021-00782-y

**Published:** 2021-12-20

**Authors:** Maryna Koskela, T. Petteri Piepponen, Maria Lindahl, Brandon K. Harvey, Jaan-Olle Andressoo, Vootele Võikar, Mikko Airavaara

**Affiliations:** 1 Institute of Biotechnology, HiLIFE, University of Helsinki, P.O. Box 56, 00014 Helsinki, Finland; 2 Division of Pharmacology and Pharmacotherapy, Faculty of Pharmacy, University of Helsinki, P.O. Box 56, 00014 Helsinki, Finland; 3 Faculty of Medicine, University of Helsinki, PO Box 56, 00014 Helsinki, Finland; 4 National Institute on Drug Abuse, IRP, NIH, Biomedical Research Center, 251 Bayview Boulevard Suite 200, Baltimore, MD 21224 USA; 5 Neuroscience Center, HiLIFE, University of Helsinki, P.O. Box 56, 00014 Helsinki, Finland; 6 Division of Neurogeriatrics, Department of Neurobiology, Care Sciences and Society (NVS), Karolinska Institutet, 17177 Stockholm, Sweden

**Keywords:** Alcohol addiction, IntelliCage, Craving, Conditional stimuli, Social interaction, GDNF, BDNF

## Abstract

**Background:**

Craving for alcohol, in other words powerful desire to drink after withdrawal, is an important contributor to the development and maintenance of alcoholism. Here, we studied the role of GDNF (glial cell line-derived neurotrophic factor) and BDNF (brain-derived neurotrophic factor) on alcohol-seeking behavior in group-housed female mice.

**Methods:**

We modeled alcohol-seeking behavior in C57Bl/6J female mice. The behavioral experiments in group-housed female mice were performed in an automated IntelliCage system. We conducted RT-qPCR analysis of *Gdnf*, *Bdnf*, *Manf* and *Cdnf* expression in different areas of the female mouse brain after alcohol drinking conditioning. We injected an adeno-associated virus (AAV) vector expressing human GDNF or BDNF in mouse nucleus accumbens (NAc) after ten days of alcohol drinking conditioning and assessed alcohol-seeking behavior. Behavioral data were analyzed by two-way repeated-measures ANOVA, and statistically significant effects were followed by Bonferroni’s post hoc test. The student’s *t-test* was used to analyze qPCR data.

**Results:**

The RT-qPCR data showed that *Gdnf* mRNA level in NAc was more than four times higher (p < 0.0001) in the mice from the sweetened alcohol group compared to the water group. Our data showed a more than a two-fold decrease in *Manf* mRNA (p = 0.04) and *Cdnf* mRNA (p = 0.02) levels in the hippocampus and *Manf* mRNA in the VTA (p = 0.04) after alcohol consumption. Two-fold endogenous overexpression of *Gdnf* mRNA and lack of CDNF did not affect alcohol-seeking behavior. The AVV-GDNF overexpression in nucleus accumbens suppressed alcohol-seeking behavior while overexpression of BDNF did not.

**Conclusions:**

The effect of increased endogenous *Gdnf* mRNA level in female mice upon alcohol drinking has remained unknown. Our data suggest that an increase in endogenous GDNF expression upon alcohol drinking occurs in response to the activation of another mesolimbic reward pathway participant.

## Background

Alcoholism is a chronic brain disorder characterized by a high risk of relapse that can occur even after a long period of abstinence [1], causing serious social and health care problems worldwide [2]. Ethanol is a known psychoactive substance with rewarding and sedative-hypnotic properties. Repeated ethanol exposure results in neuroadaptive responses [3]. The environmental contexts (cues) associated with alcohol use increase the desire to drink (craving) and can provoke relapse [4, 5]. Preclinical laboratory animal models of drug relapse and craving provided strong data suggesting that alcohol acts like other drugs of abuse by activating molecular cascade within the mesocorticolimbic system [6]. Dopamine neurons with cell bodies located in the ventral tegmental area (VTA) and projecting to the nucleus accumbens (NAc) are involved in the processing of reward-related stimuli associated with drugs of abuse [7]. Alcohol promotes dopamine release predominantly in the NAc in rodents [8] and the human brain with a preferential effect in the ventral striatum [9].

Craving is a complex set of experiences in behavior reported only by humans. However, animal models in alcohol addiction research remain critical tools to study the mechanism underlying different aspects of the disease progression [10]. Here we developed a novel model of alcohol-seeking behavior in group-housed female mice that is reproducible and cost-effective. Previously we have shown that pairing conditioned cue with extended alcohol drinking leads to an alcohol-seeking behavior after withdrawal in group-housed mice [11]. The modeling of addiction-like behavior in mice utilizing either intermittent access to increasing ethanol concentration or long-term 10–20% alcohol drinking usually takes over a month [11]. In this study, to increase the preference to drink alcohol and to form addiction-like behavior in mice, we introduced sweetened alcohol. We used saccharin (0.5%) as a sweetener, an artificial sweetener without food energy.

A growing body of data suggests that glial cell line-derived neurotrophic factor (GDNF) and brain-derived neurotrophic factor (BDNF) modulate addictive-related behavior [12–14]. GDNF is a secreted growth factor originally isolated from rat glial cell line and promotes dopamine uptake in midbrain cultures [15]. GDNF acts by first binding to co-receptor glycosyl-phosphotidylinositol-linked GDNF family receptor α1 (GFRα1), which then signals through binding to the tyrosine kinase receptor, RET [16, 17]. The ensemble of GDNF, GFRα1 and RET triggers the mitogen-activated protein kinase (MAPK)/ extracellular signal regulated kinase (ERK), phosphoinositol 3-kinase (PI3K), and phospholipase Cγ1 (PLCγ1) cascades. In rat brain, *Gdnf* mRNA was found to be highly expressed in the NAc, and its receptors mRNA (*Ret* and *Gfra1*), are highly expressed in the VTA [18].

BDNF belongs to the nerve growth factor (NGF) family and is an important mediator of neuronal maturation [19]. BDNF binds to its receptor tropomyosin-related kinase B (TrkB) that induces dimerization and autophosphorylation of TrkB. BDNF/TrkB signalling has been found to play roles in every aspect of neuronal activity, including neurogenesis, neurotransmitter release, synaptic plasticity, and axonal and dendritic morphology [20, 21]. Consequently, it activates downstream signaling via the PI3K, MAPK/ERK, and PLCγ1 pathways.

Studies show that endogenous *Gdnf* mRNA and *Bdnf* mRNA levels change differently in response to moderate or high alcohol dose exposure (reviewed in [12–14, 22]). These studies were performed on single-housed male mice or rats. While the isolation can be stressful in social species [23, 24], stress can increase alcohol drinking [25]. Furthermore, BDNF level in multiple brain regions is sex-dependent and altered in response to diverse types of stress [26, 27].

Here we aimed to study whether GDNF and BDNF overexpression in nucleus accumbens affect alcohol-seeking behavior after alcohol drinking withdrawal in female mice. We found that transduction of AAV-GDNF into nucleus accumbens suppresses alcohol-seeking behavior in female mice. Interestingly, we observed elevation of endogenous *Gdnf* mRNA level in nucleus accumbens after 10 days of voluntary sweetened alcohol consumption. To study the effect of increased endogenous GDNF expression on alcohol consumption, we analyzed alcohol intake in *Gdnf*^*wt/*hyper^ female mice [28]. In these mice, the endogenous *Gdnf* mRNA expression is enhanced and is approximately doubled in the ventral striatum. However, about a twofold increase in endogenous *Gdnf* mRNA did not affect alcohol-seeking behavior. Also, we found that, unlike AAV-GDNF, transduction of AAV-BDNF into nucleus accumbens had no effect on alcohol-seeking behavior in our model.

Cerebral dopamine neurotrophic factor (CDNF) and mesencephalic astrocyte-derived neurotrophic factor (MANF) are endoplasmic reticulum (ER) luminal proteins (reviewed in [29–31]). Both factors are known to modulate the dopamine system in the brain and are believed to be an essential part of the cellular adaptive protective pathway to cope with endoplasmic reticulum (ER) stress [32–35]. Unlike GDNF and BDNF, the role of MANF and CDNF in addiction and, particularly, in alcohol use disorder has not be studied extensively. It has been suggested that MANF can protect neurons against ethanol-induced neurodegeneration by ameliorating ER stress [36].

Here, we analyzed levels of *Manf* and *Cdnf* transcripts after alcohol drinking conditioning. Interestingly, we found that *Manf* and *Cdnf* mRNAs levels were decreased in the hippocampus, and *Manf* mRNA level was decreased in VTA after alcohol drinking conditioning. To determine whether lack of CDNF would affect alcohol-seeking behavior, we used CDNF knock out (*Cdnf*^−/−^) female mice [34]. The main phenotype of *Cdnf*^−/−^ mice, that are viable and fertile, with a normal life-span, concerns the enteric nervous system. [34]. However, we did not observe any differences in alcohol consumption or alcohol-seeking behavior in *Cdnf*^−/−^ female mice compared to wild-type littermates.

## Methods

### Experimental animals

The behavioral experiments were performed in female C57BL/6JRccHsd mice (n = 217, Envigo); *Gdnf*^*wt/*hyper^ mice [28, 37] wild type (n = 22) and heterozygotes (n = 22); and *Cdnf*^−/−^ mice [34] (n = 10) and wild type littermates (n = 10). The C57BL/6JRccHsd wild type mice were randomly assigned for the groups as follows: water group 80 mice, alcohol group 32 mice, sweetened alcohol group 32 mice, sweetened water group 9 mice, AAV-GDNF injected group 11 mice, AAV-BDNF injected group 11 mice, AAV-GFP (green fluorescent protein) injected 22 mice. We excluded from analysis 4 mice after AAV-GFP injection because mice died after the injection. For gene expression analysis of the neurotrophic factors, we used 10 mice in the sweetened alcohol group and 10 mice in the water group. The C57BL/6JRccHsd female mice arrived at the age of 8 weeks old. Mice were housed under temperature-controlled conditions at 20–22°C in a 12 h light/dark cycle with lights on at 06.00 am with ad libitum access to standard lab chow and water. The mice were individually recognized by radio-frequency identification (RFID) transponders (Planet ID GmbH, Germany). The transponders were implanted under the skin under 2.5% isoflurane anesthesia one week before experiments began. The animals were 10 weeks old at the beginning of the adaptation period in the automated cages, average 19 g of weight and grouped 8–11 mice per cage.

### Experimental apparatus

The automated IntelliCage system was used to analyze mouse behavior [11, 38]. The system (TSE, Bad Homburg, Germany) was placed in a polycarbonate cage (20.5 cm high, 58 × 40 cm top, 55 × 37.5 cm bottom, Tecniplast, 2000P, Buguggiate, Italy) [11, 13, 39, 40]. The automated cage allows performing experiments without handling the mice under fully automated conditions in the home cage environment. The cages were computer-controlled with IntelliCage Plus software performing pre-programmed experimental schedules. We used 6 automated cages simultaneously that allowed us to run experiments for big cohorts of mice with similar environmental factors.

The mouse enters the corner of the cage through a hole. All corners of the cage have an antenna that reads RFID signals and two sides with doors. When the door is open, the mouse can lick the tip of the bottle.

**Fig. 1:**
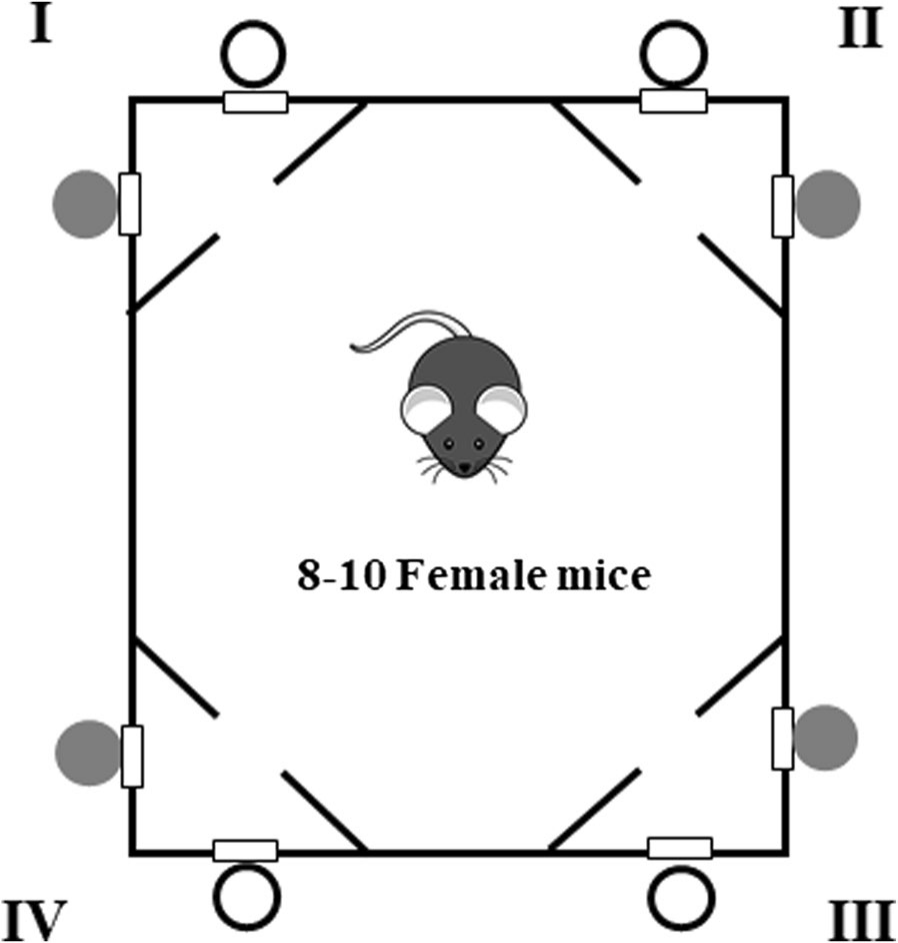
Schematic representation of the experimental settings in the automated cage used in this study. The corners of the cage are marked in Roman numerals. Sides with conditional stimulus are colored in grey, whereas non-conditional sides are colored in white

During the experiment, the computer records the following behavioral parameters: number of visits to the corner, number of nosepokes to the door, and number of licks. The nosepoke measure represents how much mice “want” to get alcohol, while the number of licks shows how much mice “like” alcohol. The schematic representation of the cages during experiments is shown in Fig. 1. A green light was used as a conditional stimulus. Four triangular red shelters (Tecniplast, Buguggiate, Italy) were placed in the middle of the cages. They were used as sleeping quarters and as a stand to reach the food. The floor of the cage was covered with a layer of bedding.

### Drugs and reagents

Ethanol (Etax A, 96% v/v; Altia, Rajamäki, Finland) was diluted into tap water. Saccharin (Sigma Aldrich, Germany) was diluted into tap water or ethanol solution.

### Behavioral procedure

The mice were randomly placed in automated cages in groups of 8–10 animals per cage with access to ethanol or water. The first week was the habituation period that consisted of the free adaptation phase (3 days, all doors in all corners were open, animals could enter and drink water in any corner) and the nosepoke adaptation phase (4 days, all doors in all corners were closed, nosepoke opened the door for 7 s). The adaptation period is required for the animal to learn to enter the corner and drink there [40]. Thereafter, the mice had access to sweetened 12% ethanol (v/v) with 0.5% saccharin or 12% ethanol, or 0.5% saccharin in the conditioned side of the corner. The schematic setup in the automated cage is presented in Fig. 1. Nosepoke opens the door in the alcohol side for 7 s and switches on a green LED light until the end of drinking.

### Alcohol withdrawal phase

After the training period, the mice were removed from automated cages, and kept in the same groups in standard home cages. For the extinction tests, the mice were brought to the automated cages for 1 h, and after each test, returned to the standard home cages.

### AAV vectors and stereotactic injections into mouse nucleus accumbens

Self-complementary adeno-associated viruses (AAV) under the control of the CMV promoter expressing human GDNF, BDNF or GFP were generated and purified as described [41, 42]. For the viral injections, animals from the alcohol groups were randomly allocated to treatment groups.

Stereotactic surgeries were done under isoflurane anesthesia (induction 4–4.5%, sustenance 2.5% isoflurane). Carprofen (5 mg/kg, s.c.) was used as a post-operative analgesic. Viral vectors were delivered bilaterally via 33-gauge needles and infused at a volume of 0.3 µl/virus and a rate of 0.1 µl /min. Coordinates for mouse nucleus accumbens (in mm, relative to bregma) were as follows: A/P 0.7, M/L ± 1.8, D/V−4.7, 10-degree angle.

### Extinction tests in automated cages

The extinction tests were performed on days 1 and/or 10 after the end of the training period between 10.00 am, and 11.00 am. The tests were performed on withdrawal days 4 and 14 on mice that received stereotactic viral injections. During the tests (1 h) experimental design was similar to the training period, except there was no liquid in the bottles. The bedding material was not changed after the conditioning period and was kept the same throughout the experiment assessing extinction for the next 10 days.

### Real-time quantitative PCR (qPCR)

A set of 20 mice were euthanized immediately after the end of alcohol drinking conditioning. Brains were rapidly extracted, frozen in − 70ºC isopentane, and stored at − 80ºC. Total RNA from frozen tissues was isolated using TRI Reagent (Molecular Research Center, USA) according to the manufacturer’s protocol. Briefly, TRI reagent was added to samples, followed by grinding with a pestle and homogenizing by pulling through a needle by a syringe. Chloroform (1/5 of TRI Reagent volume) was added to the samples, followed by 10 min incubation with subsequent centrifugation at 12,000 g for 15 min. The aqueous phase was collected and mixed with isopropanol (1/2 of TRI Reagent volume), and 1 µl of glycogen (Thermo Fisher Scientific, USA) was added to visualize the pellet. After overnight incubation at − 80ºC, the samples were centrifuged at 12,000 g for 15 min at + 4ºC, followed by washing the pellet twice with cold 75% ethanol. The pellet was air-dried and dissolved in sterile water. RNA concentration was measured by NanoDrop, and equal amounts of RNA were used for synthesizing complementary DNA (cDNA). The mRNA was converted to single-strand cDNA with DyNAmo cDNA Synthesis kit (Thermo Scientific, USA) using random hexamers and the protocol detailed by the manufacturer. qPCR was performed with TaqMan Gene Expression Assay and TaqMan Universal PCR Master Mix (ThermoFisher Scientific, USA) using Lightcycler 480 Real-Time PCR System (Roche, Switzerland). The mRNA levels of the target gene were normalized to levels of *Gapdh* as a reference gene, and quantification was performed by a ΔΔCt method. Each sample was run in duplicate. TaqMan Gene Expression Assays were: *Gdnf*, Mm00599849_m1; *Bdnf*, Mm04230607_s1; *Manf*, Mm00512511_m1; *Cdnf*, Mm00617407_m1; *Gapdh*, Mm99999915_g1.

### Immunohistochemical analysis

Mice were anesthetized with sodium pentobarbital (90 mg/kg, i.p., MebunatVet, Orion Pharma, Espoo, Finland) and transcardially perfused with PBS followed by 4% paraformaldehyde (PFA) in 0.1 M phosphate buffer, pH 7.4. Brains were post-fixed in 4% PFA at + 4°ºC and transferred to sucrose series of 10, 20 and 30% sucrose.

The brains were cut in a 40 µm thick section in a freezing microtome at − 20ºC. Free-floating sections were stained as previously described [41]. Briefly, the sections were washed in PBS and treated with 0.3% hydrogen peroxide solution. After incubation in the blocking solution (4% bovine serum albumin and 0.1% Triton X-100 in PBS) the sections were incubated with rabbit anti-GFP antibodies (1:2000, A11122, Life technologies, Bleiswijk, Netherlands) overnight at + 4ºC. Next, the sections were washed with PBS and incubated with biotinylated anti-rabbit antibodies (Vector Laboratories, Burlingame, CA, USA) and visualized with 3´,3´diaminobenzidine (Vector Laboratories, Burlingame, CA, USA). The stained sections were scanned with an automated microscope slide scanner (Pannoramic 250 Flash II, 3D Histech, Budapest, Hungary) at the BI Histoscanner core facility, HiLIFE, University of Helsinki.

### Statistical analysis

Data and graphs represent means ± SEM. GraphPad Prism (version 7.04, GraphPad Software, California, USA) was used for statistical analysis. Behavioral data were analyzed by two-way repeated-measures ANOVA and statistically significant effects were followed by Bonferroni’s post hoc test. The student’s t-test was used to analyze qPCR data. All results are presented as mean ± SEM. Significance was set at *p* < 0.05.

## Results

### Mouse model of alcohol craving after withdrawal

#### Alcohol drinking training (conditioning)

**Fig. 2:**
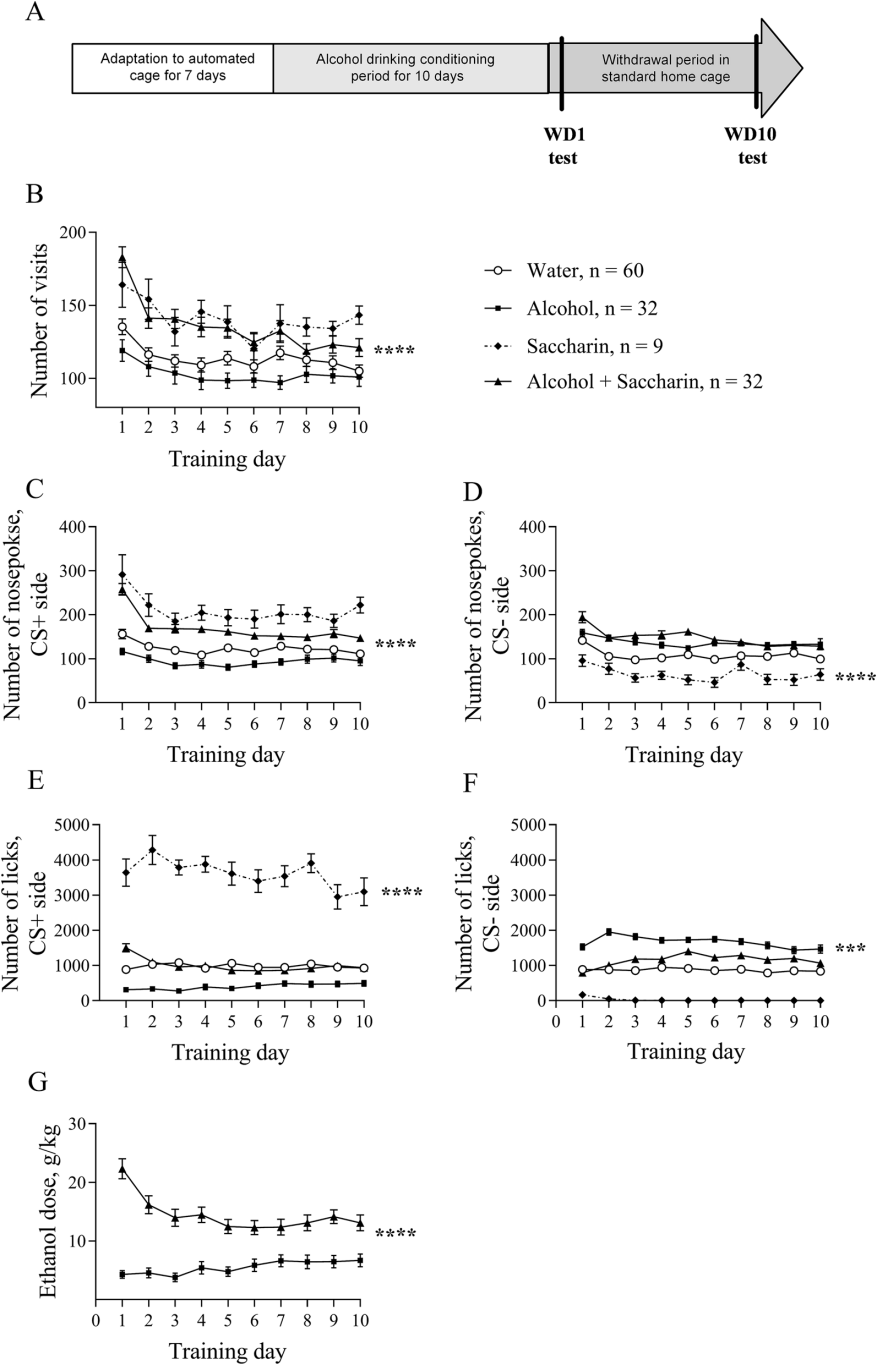
Behavioral activity in the automated cages during alcohol drinking conditioning period. **A** Schematic representation of the experimental timeline. **B** Number of visits in the corner **C** Number of nosepokes in conditioned (CS +) side. **D** Number of nosepokes in non-conditioned (CS−) side. **E** Number of licks on CS + side. **F** Number of licks in CS− side. **G** The ethanol dose that mice consumed during alcohol drinking conditioning was estimated as g/kg/24 h. ***p < 0.001, ****p < 0.0001. All means are presented with their standard errors (± SEM)

One of our aims was to define a model of alcohol craving in group-housed female C57BL/6J mice that allow them to get a craving response fast. To achieve this, we used sweetened 12% ethanol with 0.5% saccharin and compared results with unsweetened 12% ethanol, sweetened water (0.5% saccharin), and unsweetened water. We randomly allocated mice into groups. During the conditioning experiments, we had in total 60 mice in the water group, 9 mice in the saccharin group, 32 mice in the alcohol group, and 32 mice in the alcohol-saccharin group. The experimental timeline is presented in Fig. 2A. First, we assessed the behavioral activity of mice during alcohol drinking conditioning in the automated cages. Analysis of the behavioral activity during the training period revealed a significant Day effect indicating that the number of visits was different on different training days (Fig. 2B, F(9, 1161) = 25.92, p < 0.0001). Also, the number of visits differed significantly within groups during the training (Day x Training Drug interaction, F(27, 1161) = 4.13, p < 0.0001). The between-subjects analysis showed that there is a significant difference in the number of visits between groups during alcohol training (F(3, 129) = 8.08, p < 0.0001).

The within-subjects effects analysis for the number of nosepokes during the training showed a significant training Day effect in the conditioned side (CS +) (Fig. 2C, (9, 1161) = 30.84, p < 0.0001), indicating changes in the number of nosepokes during the training. Moreover, the within-subjects effects demonstrated a significance for Day x Training Drug interaction (F(27, 1161) = 5.25, p < 0.0001), showing that the number of nosepokes differs during the training within groups. Furthermore, the between-subjects analysis showed that there is a significant difference in the number of nosepokes on the conditioned side between the groups (F(3, 129) = 29.77, p < 0.0001). The within-subjects effects analysis for a number of nosepokes in the non-conditioned side also showed a significant training Day effect (Fig. 2D, F(9, 1161) = 13.82, p < 0.0001). The between-subjects analysis showed that there is a significant difference in the number of nosepokes between the groups on the non-conditioned side (F(3, 129) = 25.65, p < 0.0001).

The within-subjects effects analysis for a number of licks during the training showed a significant training Day effect in the conditioned side (Fig. 2E, F(9, 1161) = 8.429, p < 0.0001), indicating changes in the number of licks during the training. Moreover, the within-subjects effects demonstrated a significance for Day x Training Drug interaction (F(27, 1161) = 7.701, p < 0.0001), showing that the number of licks differs during the training within groups. Furthermore, the between-subjects analysis showed that there is a significant difference in the number of licks on the conditioned side between groups (F(3, 129) = 121.5, p < 0.0001). The within-subjects effects analysis for a number of licks on the non-conditioned side also showed a significant training Day effect (Fig. 2F, F(9, 1161) = 3, p = 0.0015). The between-subjects analysis showed that there is a significant difference in the number of licks in the non-conditioned corner (F(3, 129) = 68.56, p < 0.0001).

The within-subjects effect analysis for consumed alcohol dose during the conditioning showed a significant Day effect (Fig. 2G, F(9, 279) = 5.86, p < 0.0001), suggesting that consumed ethanol dose was different during conditioning. The between-subjects analysis showed that the mice consumed significantly more ethanol when drinking sweetened alcohol in comparison to the mice that drank non-sweetened alcohol (F(1, 31) = 37.54, p < 0.0001).

### Extinction test after alcohol conditioning (within-subject paradigm)

After alcohol drinking, training mice were brought back in standard home cages, and the cue-induced extinction tests were carried out on days 1 and 10 after withdrawal in the automated cages for one hour. First, we performed a test in a within-subjects paradigm. For that, we randomly took 34 mice from the water group, nine mice from the saccharin group, 16 mice from the alcohol group, and 16 mice from the alcohol-saccharin group to assess behavior on both withdrawal days.

**Fig. 3:**
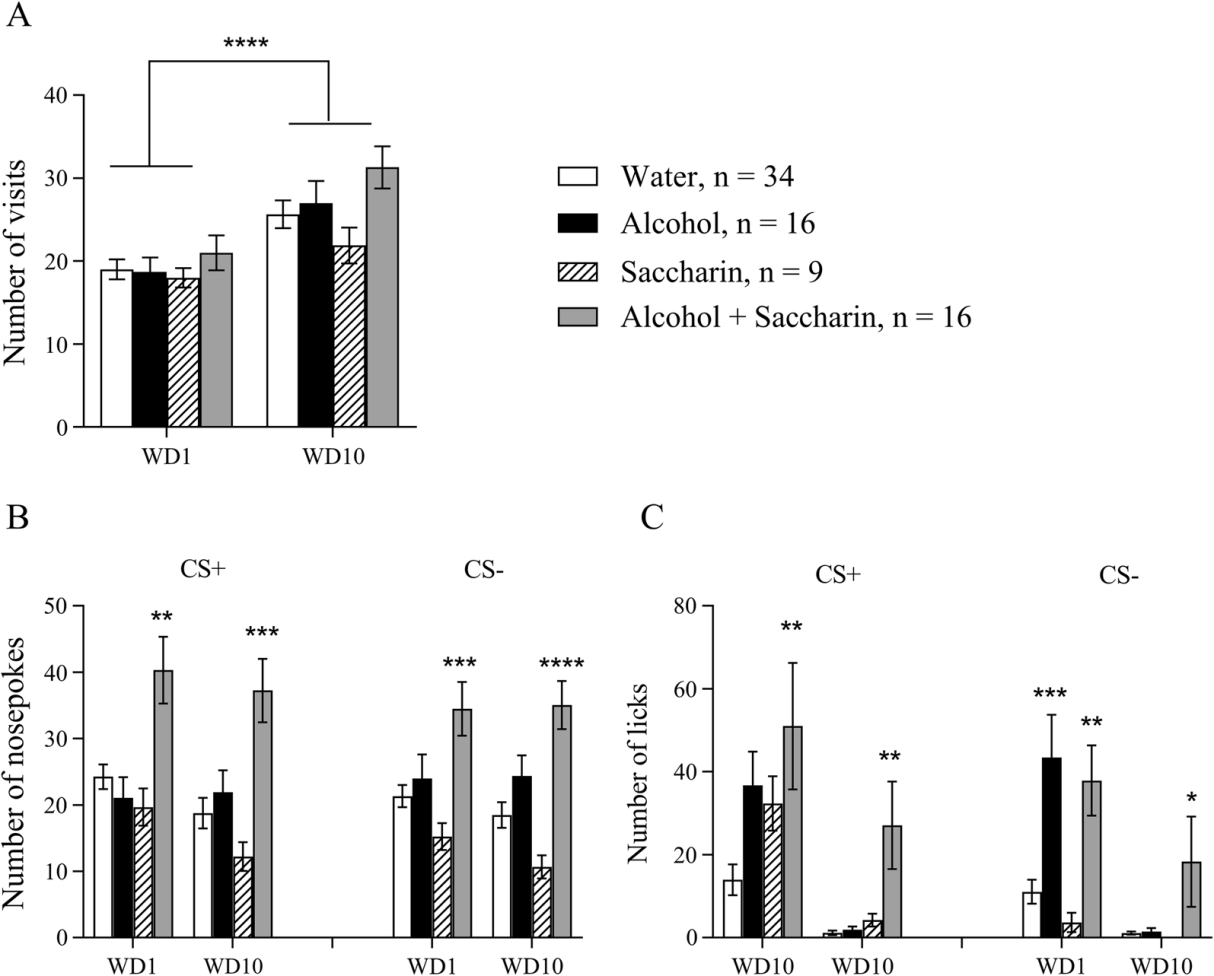
Behavioral activity in the automated cage during extinction tests after alcohol drinking conditioning period. **A** Number of visits to the corner on withdrawal day 1 (WD1) and 10 (WD10). **B** Number of nosepokes in CS + and CS− sides on WD1 and WD10. **C** Number of licks in CS + and CS− sides on WD1 and WD10. ^*^p < 0.05, **p < 0.01, ***p < 0.001, ****p < 0.0001. All means are presented with their standard errors (± SEM)

The within-subjects effect analysis of the number of visits in the conditioned corner showed a significant Day effect (Fig. 3A, F(1, 71) = 31.38, p < 0.0001), suggesting that on withdrawal day 10 mice from all groups performed more visits into the conditioned corner. However, the between-subjects effect analysis did not show a significant Training drug effect.

Then we analyzed the number of nosepokes (a measure of how much mouse wants alcohol) in CS + and CS− sides on withdrawal days 1 (WD1) and 10 (WD10). The within-subjects effect analysis showed a significant Day effect in the conditioned side (CS +) (Fig. 3B, F(1, 71) = 4.066, p = 0.0475) while in the non-conditioned side (CS−) it was not significant (Fig. 3B, F(1, 71) = 1.089, p = 0.3002). The between-subjects effects showed a significant Training Drug effect both in CS + (F(3, 71) = 10.54, p < 0.0001) and CS− (F(3, 71) = 10.75, p < 0.0001). Post hoc analysis revealed that on both WD1 and WD10 mice from Alcohol-Saccharin group performed significantly more nosepokes than mice from Water (WD1 p = 0.0012, WD10 p = 0.0001), Alcohol (WD1 p = 0.0008, WD10 p = 0.0126) and Saccharin (WD1 p = 0.0028, WD10 p = 0.0002) groups in CS + . Also Alcohol-Saccharin group performed significantly more nosepokes than Water (WD1 p = 0.0024, WD10 p < 0.0001) and Saccharin (WD1 p = 0.001, WD10 p < 0.0001) groups in CS− on both WD1 and WD10.

The within-subjects effects analysis for licks showed a significant Day effect in both CS + and CS− (Fig. 3C, F(1, 71) = 52.74, p < 0.0001 and F(1, 71) = 31.7, p < 0.0001 respectively). Also there was found a significant effects in the Day x Training Drug effect interaction in CS + and CS− (F(3, 71) = 2.763, p = 0.0482 and F(3, 71) = 6.584, p = 0.0005 respectively). The between-subjects effects showed a significant Training Drug effect in CS + and CS− (F(3, 71) = 5.53, p = 0,0018 and F(3, 71) = 6.409, p = 0.0007 respectively).

### Extinction test after alcohol conditioning (between-subject paradigm)

We next performed an extinction test in the between-subject paradigm to examine whether mice experienced incubation of craving for alcohol. To assess this, after training, we randomly assigned 16 mice from the Alcohol group with 10 mice from the control Water group and 16 mice from the Alcohol-Saccharin group with 10 mice from the control Water group to be tested on WD1 and the same amount of animals in groups to be tested on WD10. Therefore, animals that were tested on WD1 are different from those that were tested on WD10.

**Fig. 4:**
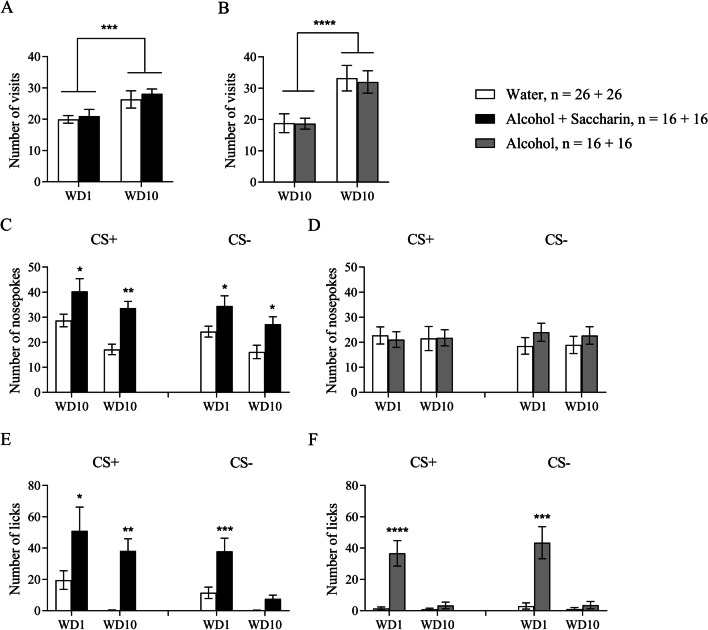
Between subject extinction tests after alcohol drinking conditioning period. **A** Number of visits of sweetened alcohol group to the corner on WD1 and WD10. **B** Number of visits of alcohol group to the corner on WD1 and WD10. **C** Number of nosepokes of sweetened alcohol group in CS + and CS− sides on WD1 and WD10. **D** Number of nosepokes of alcohol group in CS + and CS− sides on WD1 and WD10. **E** Number of licks of sweetened alcohol group in CS + and CS− sides on WD1 and WD10. **F** Number of licks of alcohol group in CS + and CS− sides on WD1 and WD10. *p < 0.05, **p < 0.01, ***p < 0.001, ****p < 0.0001. All means are presented with their standard errors (± SEM)

The within-subject effect analysis of the number of visits showed significant Day effect for Alcohol-Saccharin (Fig. 4A, F(1, 30) = 14.2, p = 0.0007) and Alcohol (Fig. 4B, F(1, 24) = 22.1, p < 0.0001) groups. However, the post hoc comparisons did not show any significant differences. The between-subjects effect analysis did not show a significant Training Drug effect in neither groups.

Next, we performed the within-subjects effect analysis of the number of nosepokes in CS + and CS− sides. The analysis revealed a significant Day effect in Alcohol-Saccharin group in CS + side (Fig. 4, F(1, 30) = 9.328, p = 0.0047) and CS− side (F(1, 30) = 5.942, p = 0.0209). The between-subjects effect analysis showed a significant Training Drug effect in CS + side (F(1, 30) = 15.56, p = 0.0004) and CS− side (F(1, 30) = 13.18, p = 0.0010). The post hoc analysis revealed significant difference in CS + on WD1 (p = 0.0304) and WD10 (p = 0.0015), and in CS− on WD1 (p = 0.0416) and WD10 (p = 0.0259). Notably, there was no significant Day effect nor Training Drug effect in Alcohol group in both CS + and CS− (Fig. 4D).

The within-subjects effect analysis of the number of licks in Alcohol-Saccharin group did not show a significant Day effect in CS + side, however there was a significant Day effect in CS− side (Fig. 4E, F(1, 30) = 17.68, p = 0.0002). The between-subjects effects analysis revealed a significant Training Drug effect in CS + (F(1, 30) = 16.85, p = 0.0003, post hoc WD1 p = 0.0341, WD10 p = 0.0087) and CS− (F(1, 30) = 13.65, p = 0.0009, post hoc WD1 p = 0.0005, WD10 p = 0.5478). Interestingly, there was a significant Day effect in both CS + and CS− sides in Alcohol group (Fig. 4F, F(1, 24) = 12.78, p = 0.0015 and F(1, 24) = 10.35, p = 0.0037 respectively). The between subjects effect analysis showed a significant Training Drug effect in both CS + and CS− sides (F(1, 24) = 9.942, p = 0.0043 and F(1, 24) = 9.366, p = 0.0054 respectively). However, the post hoc analysis revealed the significance on WD1 only (CS + p < 0.0001 and CS− p = 0.0002).

Thus, the results suggest that short term alcohol-seeking behavior—one aspect of craving—can be modeled utilizing sweetened alcohol. Moreover, there was no incubation of craving in a group of mice that consumed unsweetened alcohol while animals that consumed sweetened alcohol still showed alcohol-seeking behavior after ten days of withdrawal (Fig. 4C).

### Expression of neurotrophic factors after alcohol drinking conditioning

**Fig. 5:**
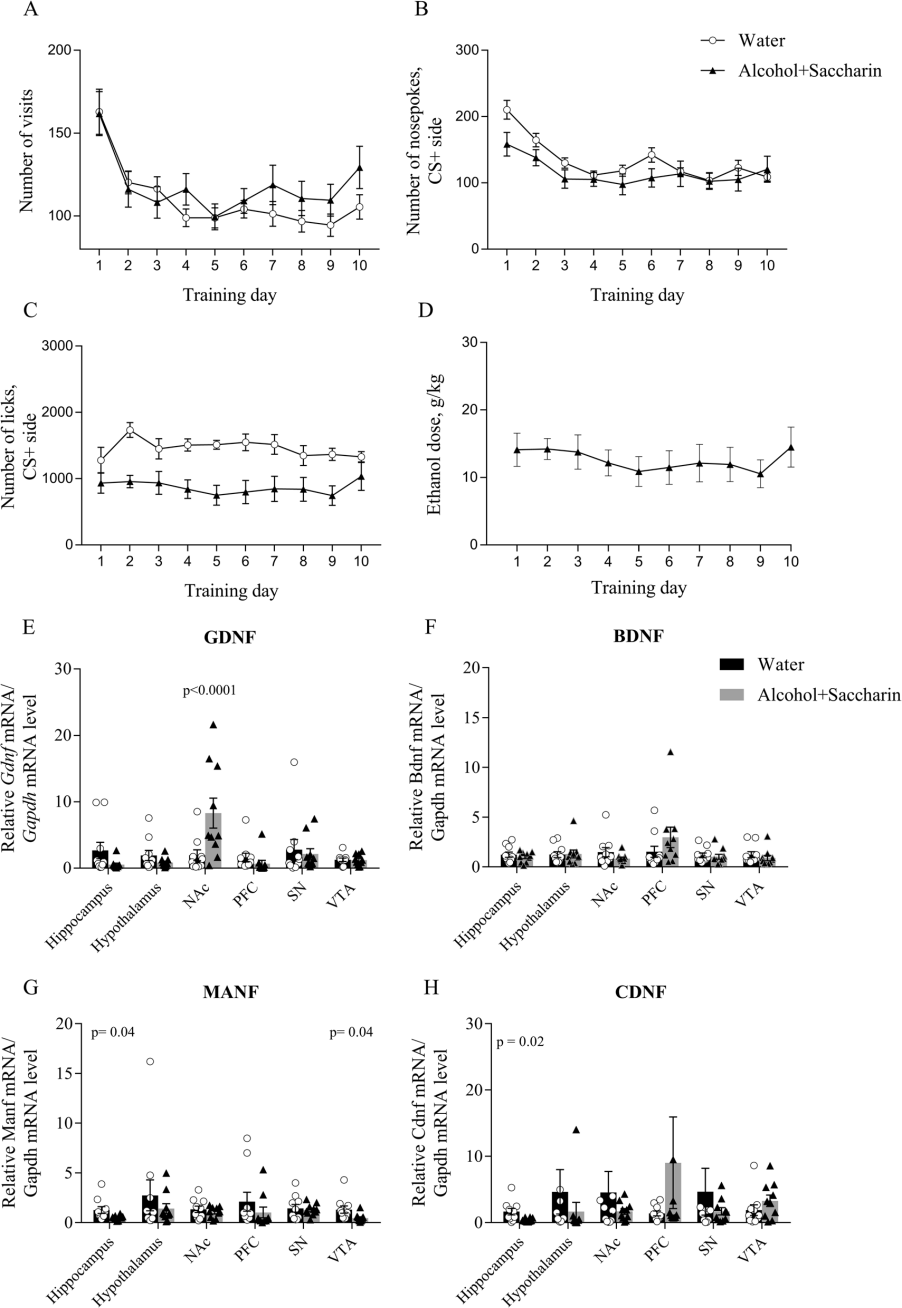
The mRNA expression of neurotrophic factors in different brain areas after sweetened alcohol drinking conditioning. **A** Number of visits in the corner. **B** Number of nosepokes in conditioned (CS +) side. **C** Number of licks on CS + side. **D** The ethanol dose that mice consumed during alcohol drinking conditioning was estimated as g/kg/24 h. **E** RT-qPCR analysis of *Gdnf* mRNA expression **F** RT-qPCR analysis of *Bdnf* mRNA expression. **G** RT-qPCR analysis of *Manf* mRNA expression. **H** RT-qPCR analysis of *Cdnf* mRNA expression

Next, we wanted to examine whether 10 days of alcohol drinking conditioning affects the expression of neurotrophic factors in different areas of the brain. Therefore, we ran an experiment with another set of animals. First, we trained mice to drink sweetened alcohol as described above. Analysis of the behavioral activity during the training period revealed a significant Day effect indicating that the number of visits was different on different training days (Fig. 5A, F(9, 81) = 20.22, p < 0.0001. The within-subjects effects analysis for a number of nosepokes during the training showed a significant training Day effect in the conditioned side (CS +) (Fig. 5B, F(9, 81) = 14.83, p < 0.0001), indicating changes in the number of nosepokes during the training. Moreover, the within-subjects effects demonstrated a significance for Day x Training Drug interaction (F(9, 81) = 2.085, p = 0.04), showing that the number of nosepokes differs during the training within groups.

The between-subjects effects analysis for a number of licks during the training showed a significant Training Drug effect on the conditioned side (Fig. 5C, F(1, 9) = 17.4, p = 0.0024), indicating that mice drank more water than sweetened alcohol during training. However, analysis of consumed alcohol (Fig. 5D) showed that at the end of the training period, mice consumed a similar ethanol dose as model mice indicating good reproducibility of the model results.

On the last day of alcohol drinking conditioning, we harvested the tissues. Next, we analyzed the expression of *Gdnf*, *Bdnf*, *Manf*, and *Cdnf* genes in the hippocampus, hypothalamus, NAc, prefrontal cortex (PFC), subtantia nigra (SN), and ventral tegmental area (VTA). Results from the RT-qPCR data showed that *Gdnf* mRNA level in NAc was more than 4 times higher (p < 0.0001) in the mice from the sweetened alcohol group compared to the water group (Fig. 5E). We did not observe significant differences in *Bdnf* mRNA expression in any of the abovementioned brain regions (Fig. 5F). Interestingly, our data showed a more than twofold decrease in *Manf* mRNA (Fig. 5G) and *Cdnf* mRNA (Fig. 5H) levels in the hippocampus (p = 0.04 and p = 0.02 respectively), and *Manf* mRNA in the VTA (p = 0.04) (Fig. 5G) after alcohol consumption.

**Fig. 6:**
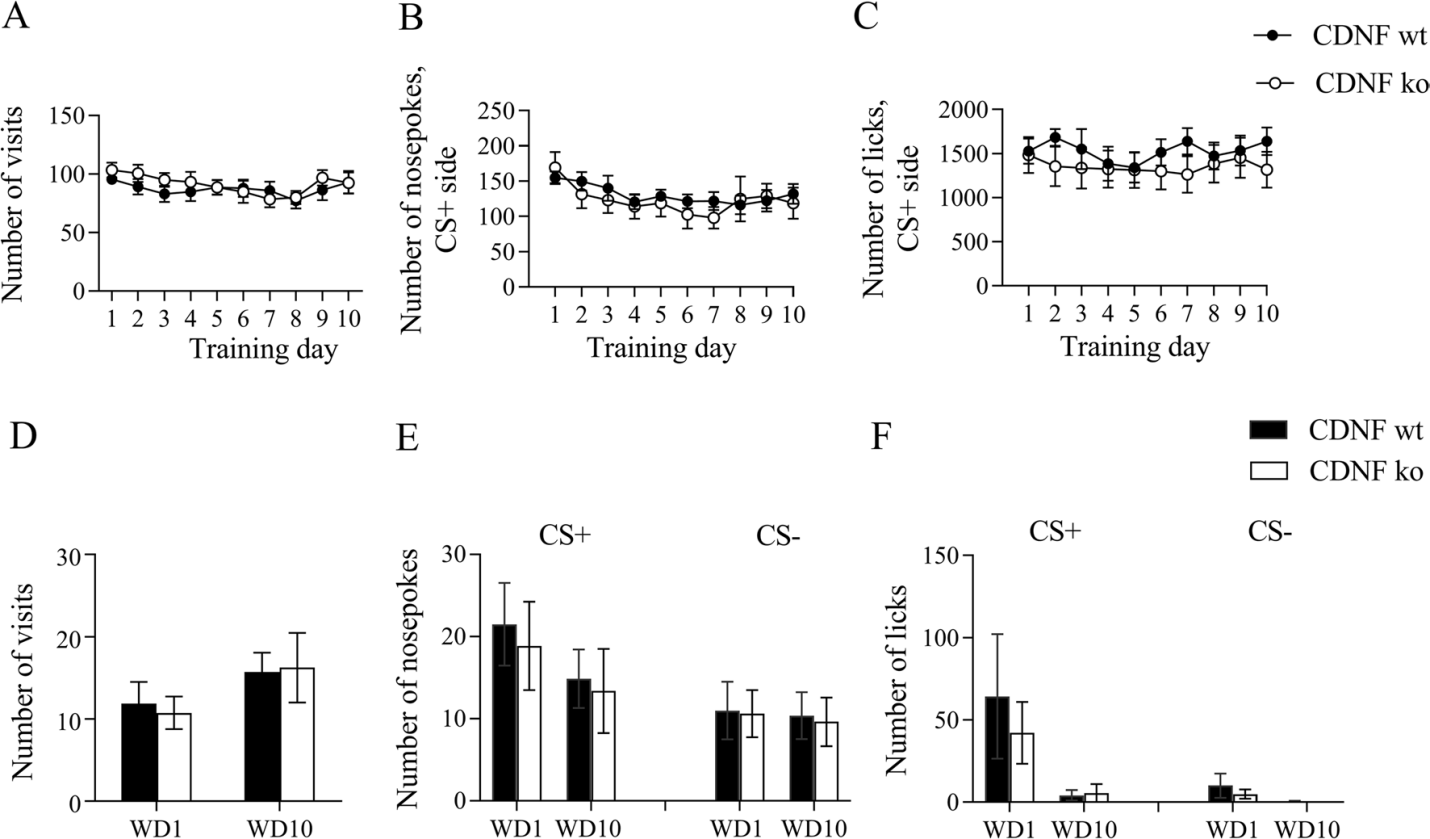
The behavioral activity of *Cdnf*^−/−^ female mice in the automated cages during alcohol drinking conditioning **A**, **B**, **C** and extinction tests **D**, **E**, **F** on WD1 and WD10. **A** Number of visits in the corner. **B** Number of nosepokes in conditioned (CS +) side. **C** Number of licks in CS + side. **D** Number of visits to the corner on WD1 and WD10. E. Number of nosepokes in CS + and CS− sides on WD1 and WD10. **F** Number of licks in CS + and CS− sides on WD1 and WD10. All means are presented with their standard errors (± SEM)

In addition, we performed behavioral analysis on CDNF knockout mice [34] to study whether the absence of CDNF would affect alcohol-drinking and alcohol-seeking behavior in female mice (Fig. 6). We did not observe any detectable effect of CDNF removal on behavior during alcohol drinking conditioning (Fig. 6A–C) and extinction test (Fig. 6D–F), suggesting that CDNF is not involved in the regulation of alcohol-drinking behaviors in female mice. However, we do not know whether MANF and CDNF overexpression in the brain could affect alcohol consumption or alcohol seeking.

### Two-fold elevation in endogenous GDNF expression does not impact alcohol craving

**Fig. 7:**
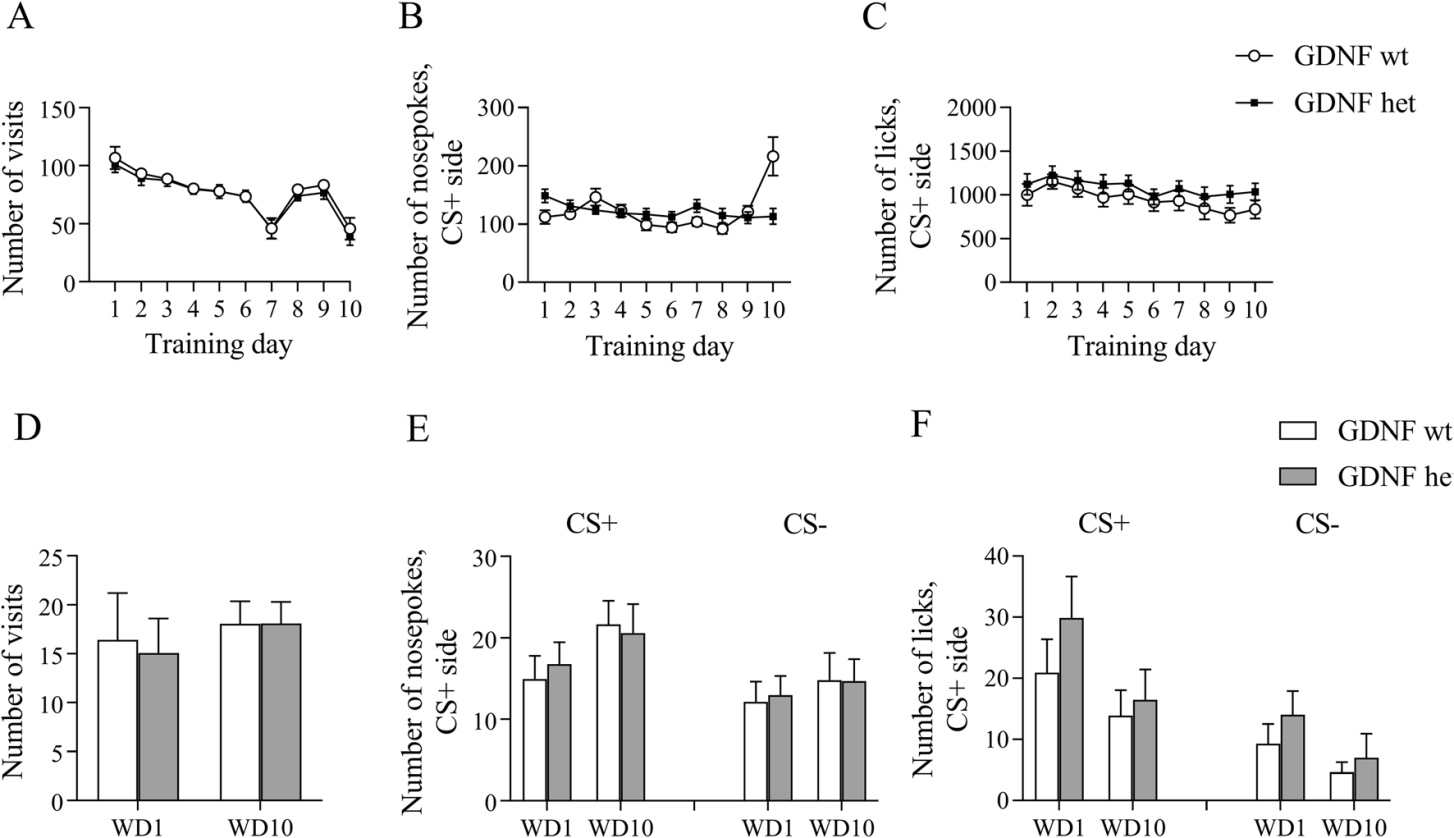
The behavioral activity of *Gdnf*^*wt/*hyper^ female mice in the automated cages during alcohol drinking conditioning **A**, **B**, **C** and extinction tests **D**, **E**, **F** on WD1 and WD10. **A** Number of visits in the corner. **B** Number of nosepokes in CS + side. **C** Number of nosepokes in CS− side. **D** Number of licks in CS + side. **E** Number of licks in CS− side. **F** Number of visits to the corner on WD1 and WD10. **G** Number of nosepokes in CS + and CS− sides on WD1 and WD10. **H** Number of licks in CS + and CS− sides on WD1 and WD10. All means are presented with their standard errors (± SEM)

The elevated GDNF level increases the number of dopamine neurons in the substantia nigra [28]. Notably, the overexpressed GDNF under the promoter not specific to GDNF-expressing neurons does not have a similar effect [43]. Therefore, we wanted to compare the effect of endogenously overexpressed GDNF in transgenic *Gdnf*^*hyper*^ female mice [28] on alcohol-seeking behavior in group-housed animals. First, we assessed the behavioral activity of mice during alcohol conditioning training in automated cages. Analysis of the behavioral activity during the training period revealed a significant Day effect indicating that the number of visits was different on different training days (Fig. 7A, F(9, 378) = 27.28, p < 0.0001). However, the between-subjects analysis did not show any significant difference in the number of visits between groups during alcohol training.

The within-subjects effects analysis for a number of nosepokes during the training showed a significant training Day effect in conditioned side (CS +) (Fig. 7B, F(9, 378) = 6.498, p < 0.0001), indicating changes in the number of nosepokes during the training. Moreover, the analysis of the within-subjects effects in CS + demonstrated a significance for Interaction (F(9, 378) = 7.876, p < 0.0001), showing that the number of nosepokes differs during the training within groups. Post hoc analysis revealed that the group of wild type mice performed more nosepokes on the last training day in comparison to a group of heterozygous mice (p < 0.0001). The between-subjects analysis did not show any significant difference in the number of nosepokes between the groups.

The within-subjects effect analysis for a number of licks during the training showed a significant Training Day effect in the CS + side (Fig. 7C, F(9, 378) = 3.75, p = 0.0002), indicating changes in the number of licks during training. The within-subjects effects did not show a significance for Day x Training Drug interaction on both sides. The between-subjects analysis did not show any significant difference in the number of licks between the groups.

After training, mice were placed in standard home cages. Next, we performed extinction tests on withdrawal days 1 and 10 after alcohol drinking conditioning. The within-subjects and between-subjects effects analysis of the number of visits in the conditioned corner did not show any significant difference (Fig. 7D). There was a significant Day effect in CS + when we performed the within-subject effect of the number of nosepokes (Fig. 7, F(1, 42) = 4.834, p = 0.0335) and the number of licks (Fig. 7F, F(1, 42) = 4.626, p = 0.0373). There were no significant changes for Interaction on both sides. Also, the between-subjects analysis did not show any significant difference in the number of licks on both sides between the groups.

### Overexpression of GDNF in nucleus accumbens suppresses alcohol-seeking behavior

**Fig. 8:**
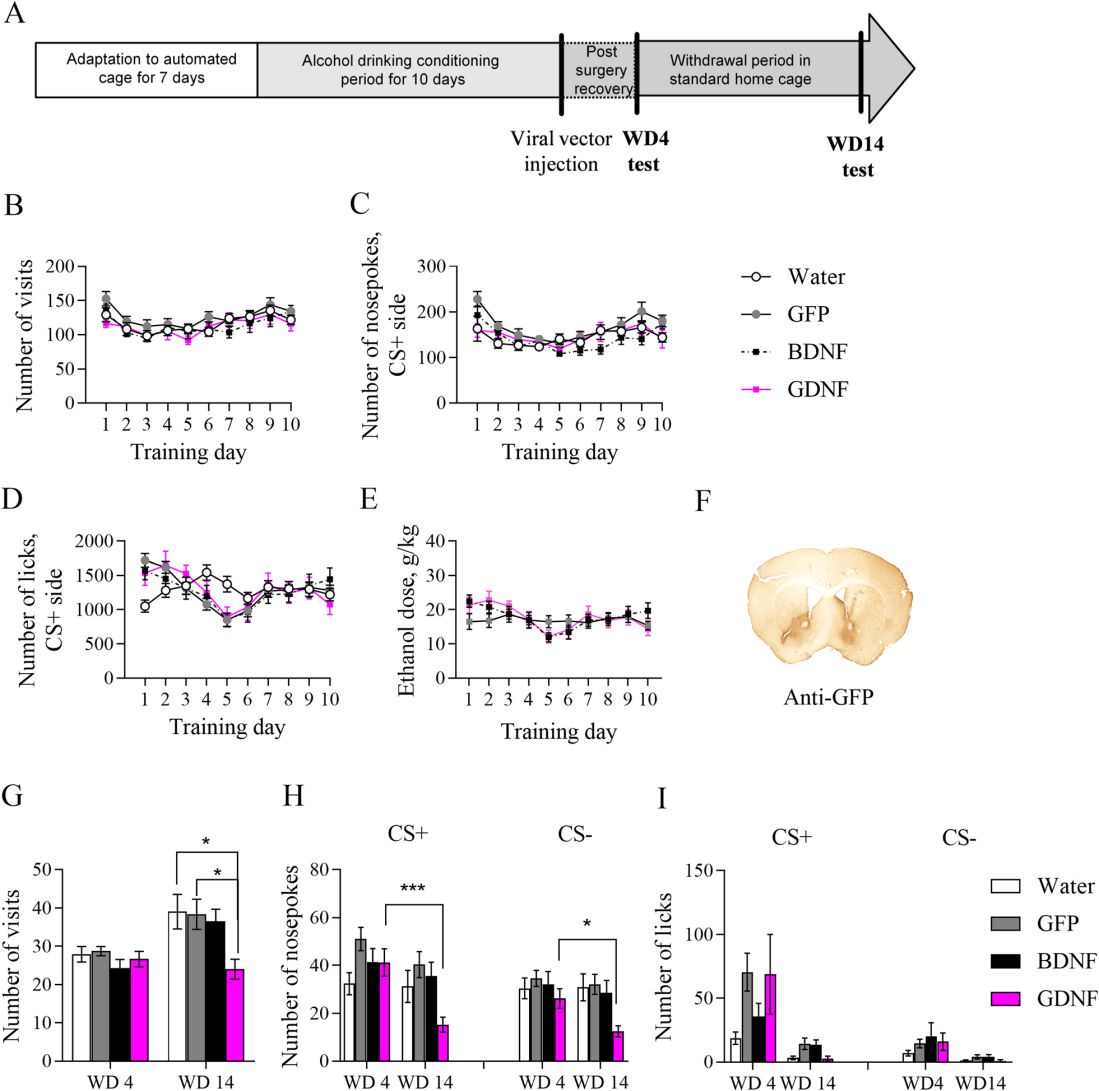
Behavioral activity in the automated cages during alcohol drinking conditioning and extinction tests after viral overexpression of GDNF and BDNF. **A** Schematic representation of the experimental timeline. **B** Number of visits in the corner. **C** Number of nosepokes in conditioned CS + side. **D** Number of licks in CS + side. **E** The ethanol dose that mice consumed during alcohol drinking conditioning was estimated as g/kg/24 h. **F** Spread of AAV-GFP viral vector in the nucleus accumbens is demonstrated by GFP immunohistochemistry. **G** Number of visits to the corner on WD1 and WD10. **H** Number of nosepokes in CS + and CS− sides on WD1 and WD10. **I** A number of licks in CS + and CS− sides on WD1 and WD10. *p < 0.05, ***p < 0.001. All means are presented with their standard errors (± SEM)

Next, we studied the effect of GDNF and BDNF overexpression on alcohol-seeking behavior in group-housed female mice. The experimental timeline is presented in Fig. 8A. First, we performed alcohol conditioning training in automated cages. We randomly allocated mice in treatment groups so that mice receiving AAV-GDNF or AAV-BDNF and AAV-GFP injections were mixed randomly for every IntelliCage. As a control, we had a cage with access to water only. Analysis of the behavioral activity during the training period revealed a significant Day effect indicating that the number of visits was different on different training days (Fig. 8B, F(9, 540) = 17.19, p < 0.0001). However, the between-subjects analysis did not show any significant difference in the number of visits between groups during alcohol training.

The within-subjects effects analysis for a number of nosepokes during the training showed a significant training Day effect in the conditioned side (Fig. 8C, F(9, 540) = 12.35, p < 0.0001), indicating changes in the number of nosepokes during the training. Moreover, the within-subjects effects demonstrated a significance for Interaction (F(27, 540) = 1.965, p = 0.0028), showing that the number of nosepokes differs during the training within groups. The between-subjects analysis did not show any significant difference in the number of nosepokes on the conditioned side between the groups.

The within-subjects effect analysis for a number of licks during the training showed a significant Training Day effect on the conditioned side (Fig. 8D, F(9, 540) = 14.77, p < 0.0001), indicating changes in the number of licks during training. Also, the within-subjects effects showed a significance for Day x Training Drug interaction (F(27, 540) = 5.147, p < 0.0001), indicating that the number of licks is different during the training within groups. The between-subjects analysis did not show any significant difference in the number of licks in the conditioned side between the groups.

We analyzed the alcohol consumption during the conditioning period. The within-subjects effect analysis showed a significant Day effect (Fig. 8E, F(9, 369) = 5.871, p < 0.0001), suggesting that the consumed ethanol dose was different during conditioning. However, the consumed ethanol dose was similar to that we observed in our model.

Then, we injected bilaterally AAV-GDNF or AAV-BDNF, or AAV-GFP, into mouse nucleus accumbens after the end of the training. AAV-GFP was used as a control. The infusion of scAAV1-GFP leads to a marked expression of GFP in the nucleus accumbens (Fig. 8F) indicating the efficient AAV delivery. For post-surgical recovery, mice were placed in standard home cages for 3 days. Next, we performed extinction tests on withdrawal days 4 and 14 after alcohol drinking conditioning.

The within-subjects effect analysis of the number of visits in the conditioned corner showed a significant Day effect (Fig. 8F, F(1, 58) = 19.07, p < 0.0001). Moreover, the post hoc analysis revealed that on withdrawal day 14, mice from Water (p = 0.0014), GFP (p = 0.0069), and BDNF (p = 0.0119) groups visited the conditioned corner significantly more in comparison to activity on withdrawal day 4. Furthermore, the post hoc analysis showed that mice from the GDNF group visited the conditioned corner on withdrawal day 14 significantly less in comparison to the Water (p = 0.0119) and GFP (p = 0.0187) groups.

The within-subject effect analysis of the number of nosepokes a significant Day effect in the conditioned side (CS +) (Fig. 8G, F(1, 58) = 15.96, p = 0.0002) as well as in the non-conditioned side (CS−) (Fig. 8G, F(1, 58) = 5.372, p = 0.024). Post hoc analysis revealed that mice from the GDNF group performed significantly fewer nosepokes on WD 14 in comparison to WD4, while no significant changes were observed in other groups. Moreover, the within-subjects effect analysis showed a significance for Day x Training Drug interaction in the conditioned side (CS +) (F (3, 58) = 3.623, p = 0.0182).

The within-subjects effects analysis for licks showed a significant Day effect in both CS + and CS− (Fig. 8H, F(1, 58) = 27.88, p < 0.0001 and F (1, 58) = 19.84, p < 0.0001 respectively). Also, analysis revealed a significant Day x Training Drug effect interaction in the CS + side (F (3, 58) = 2.875, p = 0.0438). The between-subjects effects showed a significant Training Drug effect in CS + (F (3, 58) = 3.277, p = 0.0272).

## Discussion

Craving, a persistent desire for alcohol, is one of the critical events during withdrawal from alcohol that can lead to relapse. Relapse is a major challenge to treat addiction due to a lack of understanding of what happens during withdrawal. Craving is a complex set of experiences in behavior reported explicitly by humans. A critical factor in craving is associative learning when drug consumption is paired with conditional stimuli. Though craving is challenging to model in animals with equivalence to humans, to date, data obtained in preclinical models of alcohol addiction is crucial to undercover the mechanism of disease progression.

To model alcohol craving in animals, alcohol drinking is associated with the cue, memorable stimuli. Then during withdrawal, animals undergo an extinction test. During extinction, alcohol-seeking behavior is analyzed. Despite the importance of existing animal models of craving, they face some limitations. Often animals are single housed and undergo extensive human handling. Both are stress factors for rodents. Previously we have developed an alcohol craving model in group-housed female mice with minimum human handling [11, 13]. While the model is based on a very well-established intermittent access to increasing concentrations of alcohol paradigm, it takes time and a lot of resources.

In the present study, we added a sweet taste to 12% alcohol to decrease alcohol conditioning time in group-housed female mice. We found that pairing conditional stimuli with sweetened 12% alcohol increased alcohol-seeking behavior both on WD1 and WD10. In comparison, there were no significant differences when mice drank sweetened water or 12% alcohol only. Also, after ten days of withdrawal, both groups of mice did not show incubation of craving, progressively increased a cue-induced craving after a prolonged period of withdrawal (reviewed in [44]). In addition, we did not observe any signs associated with the effect of alcohol withdrawal such as tremor, piloerection, sweating, nausea (reviewed in [45]).

The endogenous *Gdnf* and *Bdnf* levels fluctuate in response to alcohol exposure. The extensive studies on male rats associated increased levels of *Gdnf* and *Bdnf* with inhibition of excessive alcohol consumption [14]. The levels of GDNF mRNA in the NAc were unaltered in response to alcohol exposure in male rats after intermittent-access 20% ethanol [46]. However, a recent study on mixed female and male rat groups showed that GDNF mRNA expression is decreased in the VTA and increased in the NAc following withdrawal from alcohol drinking [47]. The involvement of GDNF and BNDF in addiction has been suggested based on evidence obtained from the tests of various drugs of abuse. For instance, in male rats, BDNF in the NAc promotes persistent cocaine-seeking [48], and GDNF mRNA level increases after heroin self-administration and injection of GDNF into the NAc increased craving for heroin [49]. However, a single administration of GDNF into VTA of male rats led to a rapid reduction of operant self-administration of ethanol and reduced consumption of moderate levels of ethanol [50–52]. We speculated that an increased level of GDNF and BDNF in NAc after alcohol drinking conditioning would lead to increased craving and therefore increased risk of relapse. Our data demonstrate that alcohol drinking conditioning is associated with an increased level of GDNF mRNA in the NAc of female mice. Interestingly, we did not observe significant changes in behavior in female mice with a two-fold increased endogenous GDNF mRNA level in the ventral striatum. Surprisingly, the viral overexpression of GDNF in the NAc suppressed alcohol-seeking behavior after cue-paired alcohol consumption in group-housed female mice. By contrast, in our study, the BDNF mRNA level was not changed after alcohol drinking conditioning, and the viral overexpression of BDNF in the nucleus accumbens did not affect alcohol-seeking behavior in group-housed female mice. Different studies show that BDNF expresses differently in various brain regions between female and male animals, and these differences vary among the species [26]. Therefore, for future studies to make comprehensive conclusions on neurotrophic factors mechanism of action during alcohol consumption and withdrawal, it would be essential to perform comparative studies between sex and species. In addition, it has been shown that endogenous and exogenous GDNF have different effects on tyrosine hydroxylase levels [41, 53] and dopamine homeostasis [54, 55]. Therefore, the analysis of the differential effects of endogenous and exogenous GDNF on midbrain dopamine neurons during alcohol withdrawal should be a subject of future research.

We also analyzed *Manf* and *Cdnf* mRNAs levels after 10 days of sweetened alcohol drinking. The cytoprotective role of MANF and CDNF have been demonstrated in different conditions. However, the role of MANF and CDNF in addiction and, particularly, in alcohol use disorder is not clear. Interestingly, we found that *Manf* and *Cdnf* mRNAs levels were decreased in the hippocampus, and *Manf* mRNA level was decreased in VTA after alcohol drinking conditioning. To determine whether lack of CDNF would affect alcohol-seeking behavior, we used *Cdnf*^−/−^ female mice. The main phenotype of *Cdnf*^−/−^mice, that are viable and fertile, with a normal life-span, concerns the enteric nervous system. [34]. However, we did not observe any differences in alcohol consumption or alcohol-seeking behavior in *Cdnf*^−/−^ female mice compared to wild-type littermates.

The sex is an essential biological factor, particularly in addiction research [56, 57]. Reported experimental data on the mechanism of action of neurotrophic factors in alcohol addiction-related behavior is mostly obtained from male rodents and is often extrapolated to females without experimental evidence. Moreover, usually, animals are single-housed, that is a stressful factor for social species like rats and mice [23, 24]. Also, social interaction is a rewarding stimulus for rats [58], and they choose to socialize over methamphetamine and heroin use [59, 60]. In addition, expression data of neurotrophic factors from female mice is limited [26, 27]. Although the use of female mice in IntelliCage is recommended [39], in our study, the use of female mice is a matter of practicability and feasibility. Nevertheless, our data are first to investigate the expression of different neurotrophic factors in group-housed female mice. However, further studies are necessary to determine whether there are sex-dependent differences in neurotrophic factor expression during alcohol consumption and withdrawal in group-housed animals.

Additional studies that explore what types of neurons in the nucleus accumbens activated by GDNF overexpression are involved in mediating the decreasing craving after withdrawal from alcohol drinking are also warranted. Moreover, to better understand the mouse behavior, it would be important to tackle the problem of blood alcohol levels (BAL) measurements in this model. At the moment, it is a very challenging task. Taking blood samples daily during alcohol conditioning would be an added stressor for animals while our study focuses on the stress- and handling-free mouse model. Moreover, the amount of blood that is possible to get, for example, from the tail vein is a limiting factor because BALs are almost undetectable. Therefore, such measurements can be considered only at the end of the alcohol drinking period when mice are sacrificed to get enough blood.

## Conclusion

Taken together, the effect of increased endogenous *Gdnf* mRNA level upon alcohol drinking has remained unknown. *Gdnf*^*hype*r^ mice provided us unique opportunity to analyze this. Our data revealed that about two-fold increase in endogenous *Gdnf* mRNA expression does not affect alcohol consumption in female mice. This suggests that an increase in endogenous GDNF expression upon alcohol drinking occurs in response to the activation of another mesolimbic reward pathway participant.

## Data Availability

The datasets used and/or analysed during the current study are available from the corresponding author on reasonable request.

## Abbreviations

AAV: Adeno-associated virus
BDNF: Brain-derived neurotrophic factor
CDNF: Cerebral dopamine neurotrophic factor
CS: Conditional side
ER: Endoplasmic reticulum
ERK: Extracellular signal regulated kinase
GDNF: Glia cell line-derived neurotrophic factor
GFP: Green fluorescent protein
GFR: Glycosyl-phosphotidylinositol-linked GDNF family receptor α1
MANF: Mesencephalic astrocyte-derived neurotrophic factor
MAPK: Mitogen-activated protein kinase
NAc: Nucleus accumbens
PI3K: Phosphoinositol 3-kinase
PLCγ1: Phospholipase Cγ1
RET: Tyrosine kinase receptor
WD: Withdrawal day
